# Supplementary material for: Confinement anisotropy drives polar organization of two DNA molecules interacting in a nanoscale cavity
Source: Nat Commun. 2022 Jul 28;13:4358. doi: 10.1038/s41467-022-31398-x (PMC9334635; doi:10.1038/s41467-022-31398-x)
Supplement: Supplementary file 1 — Supplementary information [file 41467_2022_31398_MOESM1_ESM.pdf]

Supplementary Information for "Confinement Anisotropy Drives Polar Organization of Two DNA Molecules Interacting in a Nanoscale Cavity"

Z. Liu, X. Capald, L. Zeng, Y. Zhang, R. Reyes-Lamothe and W. Reisner

Supplementary Note 1

In this section we present details for how the dwell time in a given polar-proximal state was extracted for the system containing two λ-DNA molecules. While we can fit directly to the histogramed dwell-time data shown in Fig. **3c** in the manuscript, we believe it is preferable to fit to the corresponding cumulative dwell-time histogram. The reason is that results obtained from fitting to the histograms may be biased by the particular bin size selected to create the histogram, while the cumulative histogram does not require a particular bin size to be determined. Supplemental Fig. 1 shows results of fitting the cumulative of a single exponential to the cumulative dwell-time histogram for all cavities investigated (using a least-squares method). The mismatch between the fitted model and cumulative data at high dwell times arises from the low statistics at very long dwell times.

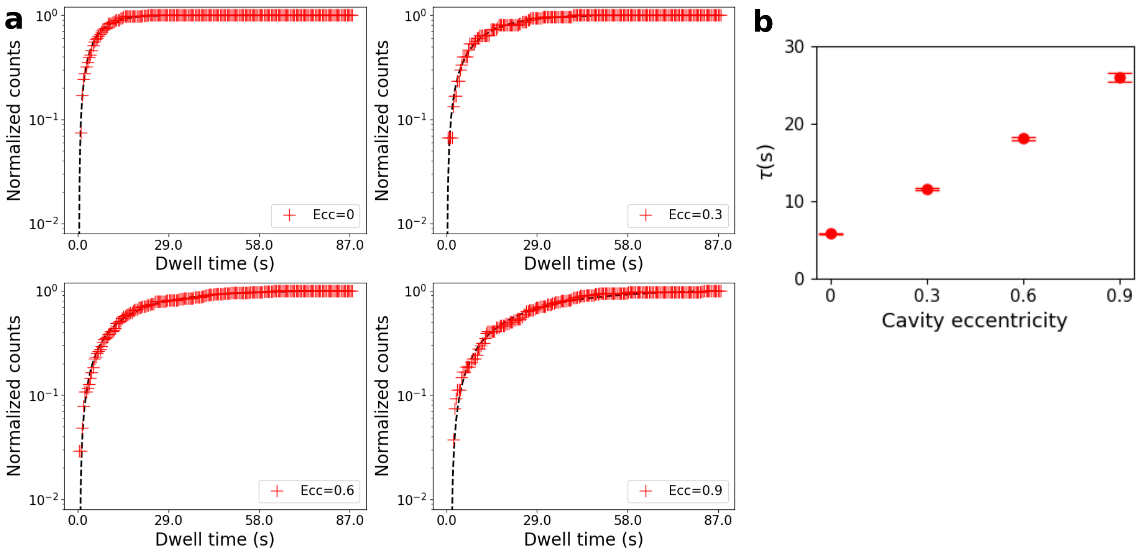

Supplementary Fig. 1: Cumulative dwell time fitting for the two DNA confinement. **a**. Results of cumulative fitting for evaluation of the dwell-time τ of the two-chain system in a given polar-proximal state. The blue points give the cumulative dwell-time histogram; the dashed red line the exponential fit. Note that the cumulative histograms have been normalized by the total number of counts. **b**. Obtained dwell-time versus cavity eccentricity. The error bars denote the fitting covariance.

Supplementary Note 2

We use an open-source finite element PDE solver, FreeFEM, to solve the numerical model for the T_4-DNA concentration in an elliptical cavity. The PDE to be solved is:

$$E\Psi = -\xi^2 \Delta\Psi + \Psi^3, \tag{1}$$

where E is an eigenvalue related to the total system free energy, Ψ is the T_4-DNA concentration profile and ξ is the correlation length. The value of ξ does not change the concentration profile of the ground state solution visibly when ξ is on the order of $1\,\mu$m. ξ of a T_4-DNA is measured to be $3.96\,\mu$m[2]. Dirichilet boundary conditions ($\Psi = 0$) are applied the ellipse edges. We address the non-linear Ψ^3 term by a classical iteration method. The PDE is linearized via:

$$E\Psi_{k+1} = -\xi^2 \Delta\Psi_{k+1} + \Psi^2_{k-1,k}\Psi_{k+1}, \tag{2}$$

where $\Psi_{k-1,k} = \frac{1}{2}(\Psi_{k-1} + \Psi_k)$ and Ψ_k is the solution for the kth iteration. The termination condition is $\int(\Psi_{k+1} - \Psi_{k-1,k})^2 d\Omega < \epsilon$, where ϵ is set to $1e^{-16}$ in our case. The square of the ground-state of the solution is the concentration profile of T_4-DNA.

Supplementary Note 3

To show that the plasmid circumferential ring-shaped distribution and pole preference are not sensitive to the choice of the wall depletion function, we investigate the Weeks-Chandler-Andersen (WCA) potential[4] as an alternative model for the plasmid-boundary interaction. The WCA potential is formulated as:

$$V_{\mathrm{WCA}}(r) = \begin{cases} 4\epsilon\ [(\frac{\sigma}{r})^{12} - (\frac{\sigma}{r})^{6} + \frac{1}{4}] & \text{if } r < \sigma 2^{\frac{1}{6}} \\ 0 & \text{otherwise} \end{cases}$$

where σ characterizes the effective distance over which the potential decreases from the wall and ϵ characterizes the strength of the potential. By replacing the exponential function in Eq. (1) in the manuscript with the WCA function, we fit the model by the same protocol (maximize the cosine similarity with Nelder-Mead alogrithm[3]). The result is shown in Fig. 2. The model matches our experiment qualitatively with the plasmid circumferential ring-shaped distribution and pole preference clear. The position of the segregation peak is captured by our model.

The WCA potential is similar to a hard-wall potential, which quantitatively over estimates the plasmid-boundary interaction and provides an upper bound for the radius of gyration of the plasmid. The parameter $\sigma = 202 \pm 2\,\mathrm{nm}$, extracted from cosine similarity fitting. The modeled position distribution and comparison with experiment are shown in Fig. 2**a**-**e**.

The radius of gyration of the plasmid extracted from the model with WCA wall potential gives a upper bound of the actual radius. As shown in Fig. 2**f**, for exponential wall potential model, when $r = r_b$, the plasmid will have a potential energy more than $15k_BT$. On the contrary, for model with WCA wall potential function, the plasmid will have a potential energy less than $1k_BT$.

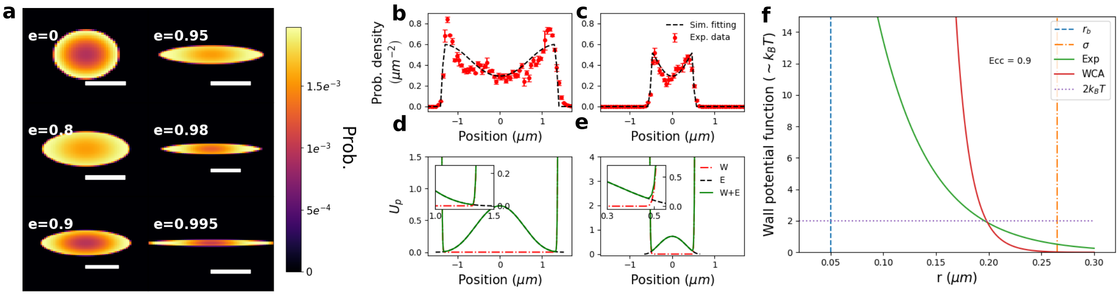

Supplementary Fig. 2: Simulation results from the model with WCA wall potential. **a**. Fitted position distribution of the confined plasmid in cavities containing a single T_4-DNA molecule with eccentricity ranging from $e = 0$ to $e = 0.995$. The scale bars from $e = 0$ to $e = 0.995$ correspond to $1\,\mu$m, $1\,\mu$m, $1.2\,\mu$m, $1.2\,\mu$m, $1.6\,\mu$m and $1.6\,\mu$m respectively. **b**,**c**. Plasmid probability density along the major (**b**) and minor (**c**) axis for $e = 0.9$. Experimental data are shown as red points, with the error bars showing the standard error of the mean of the binned counts (n=3 bins for each point). The center of the error bars denote the mean value of the 3 binned counts. Black dashed lines indicate the resulting fitted model plasmid probability density. **d**,**e**. Cross-sectional slices of the predicted potential along the major (**d**) and minor (**e**) axis for $e = 0.9$. The red dot-dashed line indicates the wall-potential; the black dashed line indicates the exclusion potential arising from the T_4-DNA; the green solid line indicates the superposition of both potentials. Note that the WCA wall potential is similar to a hard-wall potential, producing a sharper potential profile at the edge compared with the exponential wall-potential. **f**. Comparison of fitted WCA and exponential wall potential model profile. The red solid line indicates the WCA wall potential. The green solid line indicates the exponential wall potential. The blue dashed line indicates $r = r_b$. The orange dot-dashed line indicates $r = \sigma$. The purple dotted line indicates wall potential at $2\,k_BT$.

Supplementary Note 4

Here we compare the experimental and model plasmid distribution in greater detail. Overall the simulation matches our experimental results qualitatively, with the plasmid circumferential ring-shaped distribution and pole preference evident (see Fig. 4**b** and Fig. 5**a** in manuscript). To provide a more quantitative comparison, we present the cross-section of the experimental and theoretical plasmid probability distribution along the major- and minor- axis of the ellipse (see supplementary Fig. 3). Note that the positions of the peak plasmid probability agree well with the fitted model. Moreover, the potential difference ΔF between the peak and valley of the free energy barrier, which is reflected by the minor-axis cross-section of the plasmid distribution (see supplementary Fig. 3**d**), decreasing as the eccentricity increases, leading to the break of the distribution ring in the middle. We also show the same results for a model generated using the WCA wall potential (see supplementary Fig. 4).

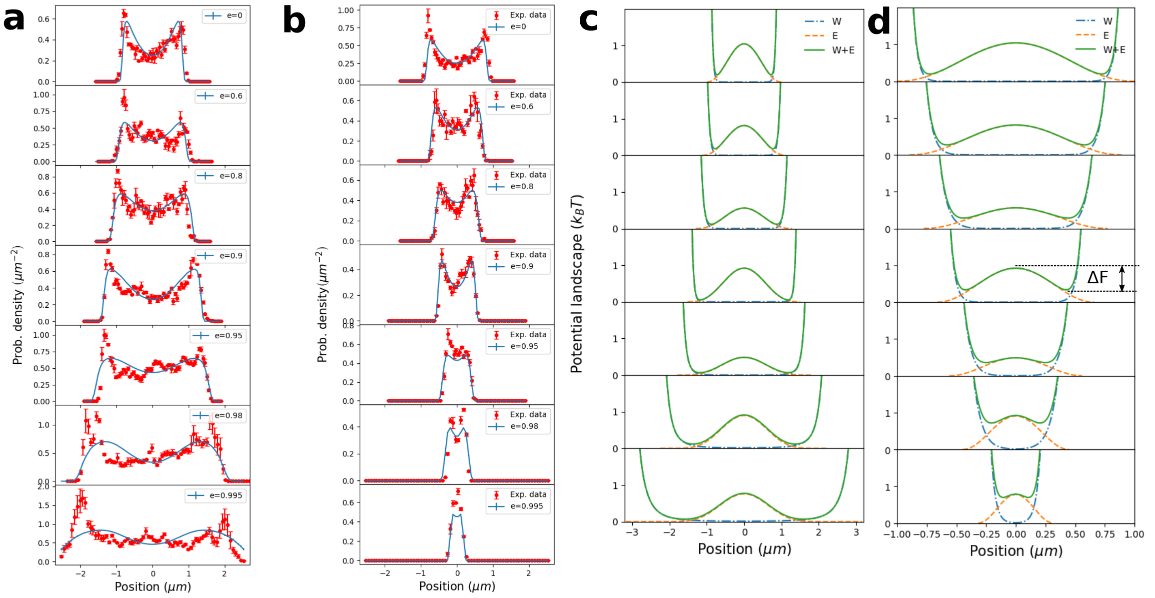

Supplementary Fig. 3: The cross-sections of the plasmid distribution and the free energy landscape from experiments and simulations with the exponential wall potential. **a**. Probability density for experimental and model plasmid position along the major axis cross-section. **b**. Probability density for experimental and model plasmid position along the minor axis cross-section. The box over which the cross-section is averaged is three bins wide ($\sim$ 150 nm). The error bars denote the standard error of the mean of the binned counts (n=3 bins for each point). The experimental data is represented by red solid circles with error bar indicating the standard error of the mean for the three adjacent bins. The blue solid line represents the simulation data for the same sampling area as the experimental data. **c**. Potential landscape obtained from simulation for a cross-section taken along the cavity major axis. **d**. Potential landscape obtained from simulation for a cross-section taken along the cavity minor axis. The blue dashed-dot line represents the wall potential. The orange dashed line represents the volume exclusive potential arising from the T_4-DNA. The green solid line gives the total potential (wall plus DNA).

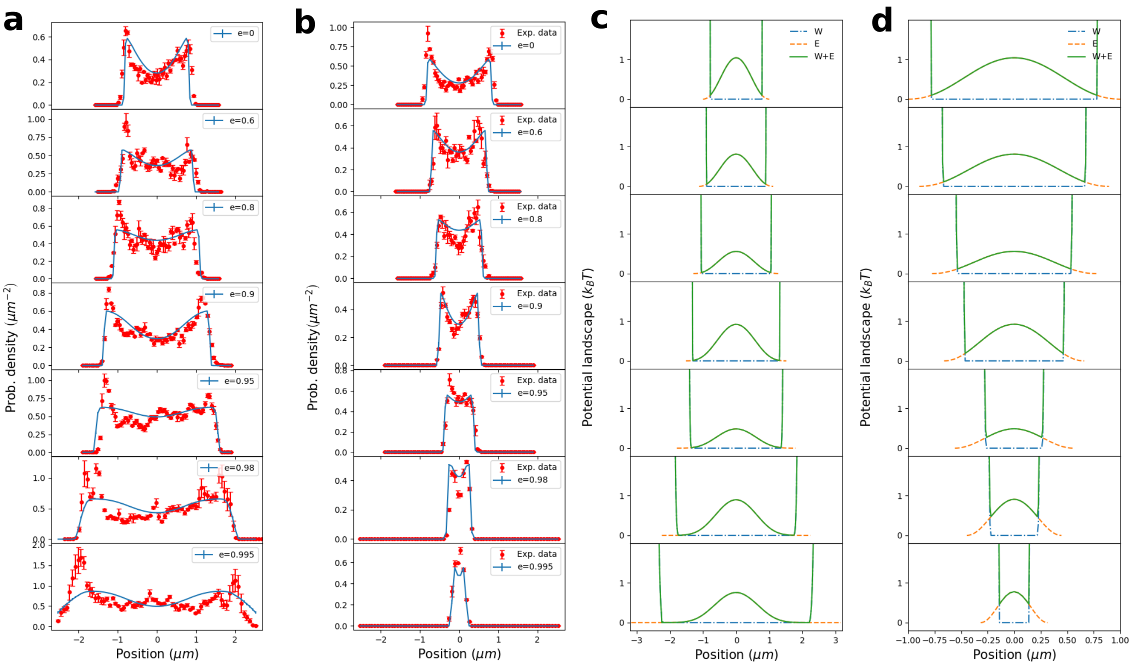

Supplementary Fig. 4: The cross-sections of the plasmid distribution and the free energy landscape from experiments and simulations with the WCA wall potential. **a**. Probability density for experimental and model plasmid position along the major axis cross-section. **b**. Probability density for experimental and model plasmid position along the minor axis cross-section. The box over which the cross-section is averaged is three bins wide ($\sim$ 150 nm). The error bars denote the standard error of the mean of the binned counts (n=3 bins for each point). The experimental data is represented by red solid circles with error bar indicating the standard error of the mean for the three adjacent bins. The blue solid line represents the simulation data for the same same sampling area as the experimental data. **c**. Potential landscape obtained from simulation for a cross-section taken along the cavity major axis. **d**. Potential landscape obtained from simulation for a cross-section taken along the cavity minor axis. The blue dashed-dot line represents the wall potential. The orange dashed line represents the volume exclusive potential arising from the T_4-DNA. The green solid line gives the total potential (wall plus DNA).

Supplementary Note 5

We also fit to a cumulative histogram to extract the polar dwell time of the plasmid while confined with T_4-DNA. To account for the short and long-time scale observed in the histogram (see Fig. 6**a** in manuscript), we use a double exponential model. This describes our experimental data well(see Fig. 5).

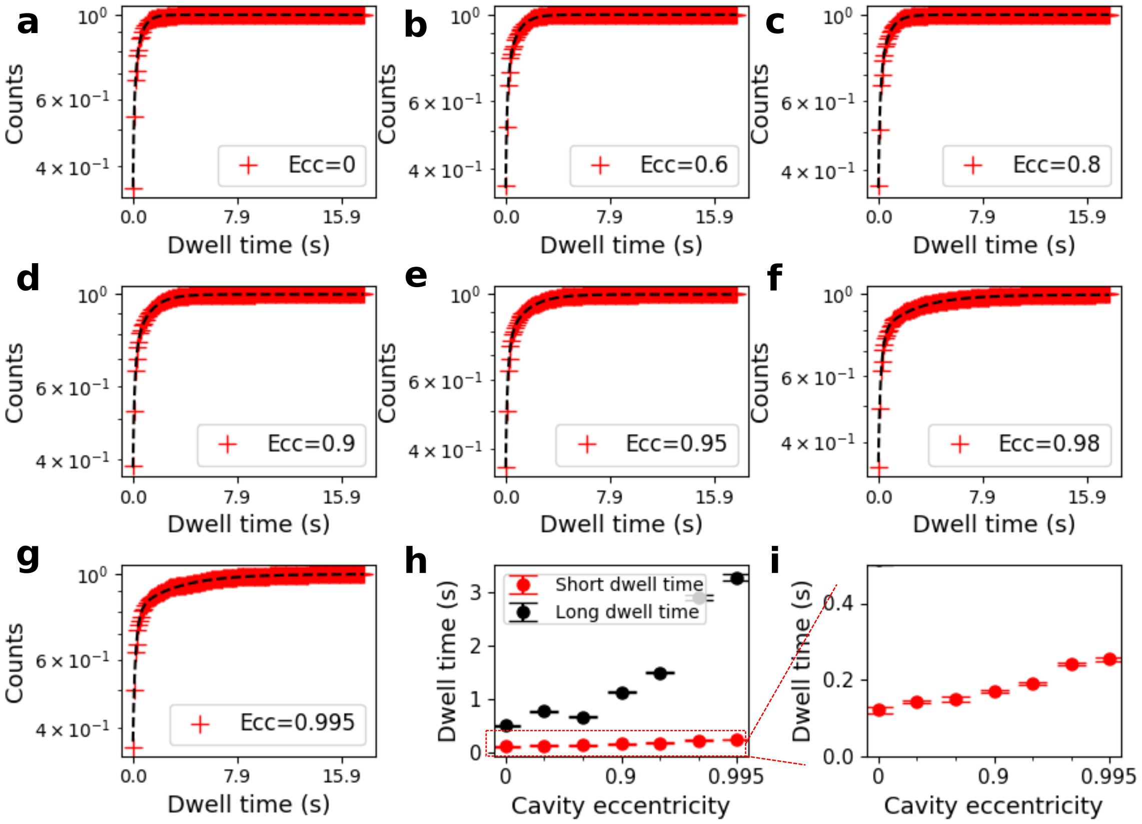

Supplementary Fig. 5: The cumulative dwell time fittings for the plasmid–T_4-DNA experiments. **a-g**. We extract the plasmid polar dwell-times by fitting the cumulative curve for a double exponential model to the cumulative plasmid dwell-time histogram. Note that the cumulative histograms have been normalized by the total number of counts. The black crosses are the experimental data. The red dashed line is the fitted double-exponential model. **h-i**. The two different time-scales extracted from the double-exponential fitting of the dwell time histogram. The black solid circle indicates the long dwell time and the red solid circle indicates the short dwell time. The errorbars are extracted from the fitting covariance.

We further investigate the events yielding the two different time scales. To do this, we plot separately the histogram of the plasmid position for events corresponding to the two different time-scales. For short-time scale events, we show the positions of plasmids which stay inside the pole region less than 0.3 s (this upper bound corresponds to the short average dwell-time extracted from double exponential fitting). For the long-time events, we show the positions of plasmids which stay inside the pole region longer than the corresponding long average dwell time extracted from double exponential fitting. The results are shown in Fig. 6. Note that the events corresponding to the short-time scale are concentrated at the border of the pole region (e.g. $|x| = l/3$), while the long-time scale events are concentrated at the free-energy pocket created by the T_4-DNA and the cavity wall.

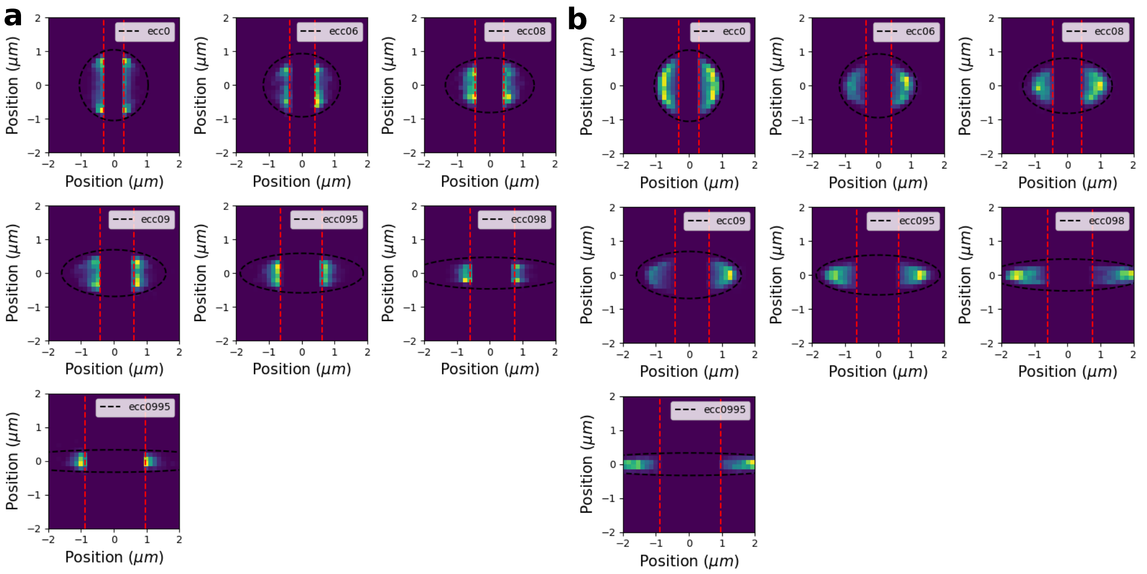

Supplementary Fig. 6: Spatial distribution of the short dwell time events and long dwell time events. **a**. Plasmid position distribution for events corresponding to the short time scale (dwell time less than 0.3 s). **b**. Plasmid position distribution for events corresponding to the long time scale (dwell time longer than the long average dwell time extracted from double exponential fitting).

Supplementary Note 6

We extract the mean-squared displacement (MSD) of the plasmid confined in the cavity with the T_4-DNA. The MSD is shown in Fig. 7**a** and is separated into its two orthogonal components along the major-axis (Fig. 7**b**) and the minor-axis (Fig. 7**c**) of the cavity. Qualitatively, the MSD is dominated by the major-axis component due to its larger spatial extent compared with the minor-axis. The major-axis component of the MSD does not greatly change in the short-time regime (less than 1 s) while the minor-axis component shows a decreasing diffusivity as the eccentricity increases.

The plasmid undergoes sub-diffusion indicated by the scaling exponent $\alpha < 1$, where $\mathrm{MSD}(\tau) \sim t^{\alpha}$. By fitting the MSD for lag time less than 1 s with the least-squared method, we extract α and the result is shown in Fig. 7**d**. We denote the corresponding exponents extracted from the orthogonal components as α_{major} and α_{minor}. We observe that α is dominated by α_{major} as the increasing of the eccentricity. α_{major} does not have a obvious trend with the eccentricity (Fig. 7**b**), while α_{minor} shows a monotonic decreasing as the eccentricity. The decreasing of the diffusivity agrees with the stronger confinement in the minor-axis direction, inducing a higher average T_4-DNA concentration in minor-axis direction.

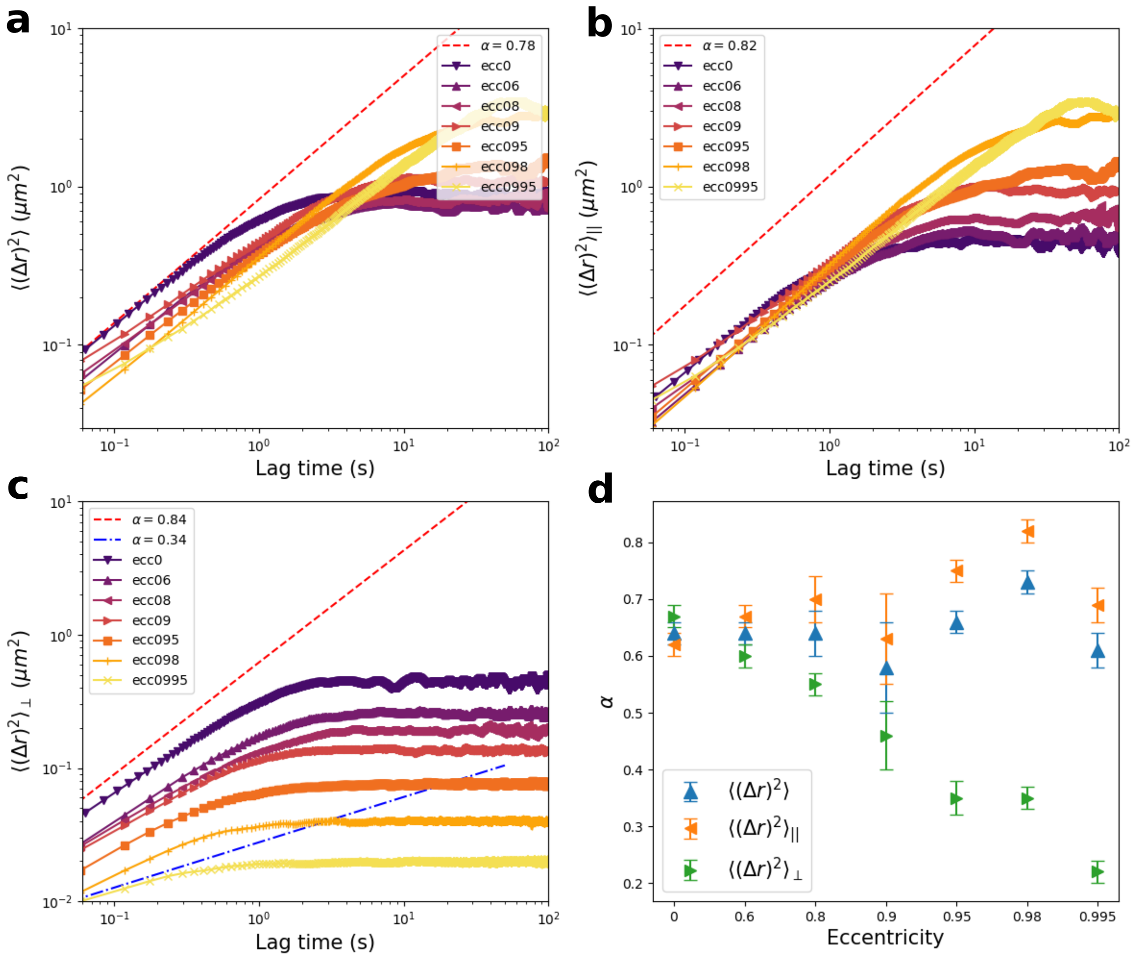

Supplementary Fig. 7: Ensemble-averaged MSD of the plasmid confined in the cavity. **a**. The full MSD of the confined plasmid. **b**. The MSD along the cavity major-axis. **c**. The MSD along the cavity minor-axis. The red dashed lines in (**a**,**b**) indicate the α extracted from the fitting averaged over all curves for varying eccentricity. The red dashed line in **c** indicates the scaling exponent for the cavity with eccentricity 0 and the blue dashed line indicates the scaling exponent for the cavity with eccentricity 0.995. **d**. Scaling exponents extracted from fitting to the MSD results. The blue triangles indicate the exponent for full MSD. The orange triangles give the scaling exponent for the MSD major-axis component. The green triangles give the scaling exponent for the minor-axis component. The error bar shows the standard error of the mean of α over captured videos ($n = 5$ for $e = 0$, $n = 7$ for $e = 0.6$, $n = 8$ for $e = 0.8$, $n = 9$ for $e = 0.9$, $n = 15$ for $e = 0.95$, $n = 9$ for $e = 0.98$, $n = 16$ for $e = 0.995$).

Supplementary Note 7

To determine if the sub-diffusive behavior of the plasmid arises from the structure of the potential landscape, we performed a Brownian dynamics simulation as a comparison. In this simulation, the 2D free energy landscape was extracted from $F_{\mathrm{CM}}(\mathbf{r}) = -k_B T \log P_{\mathrm{plasmid}}(\mathbf{r})$, where $P_{\mathrm{plasmid}}(\mathbf{r})$ is the fitted probability distribution of the plasmid position in the cavity (i.e. shown in Fig. 5**a** in the manuscript). We then modeled the plasmid's dynamics as Brownian diffusion in potential landscape $F_{\mathrm{CM}}(\mathbf{r})$, which was implemented by performing a random walk in this landscape. Each plasmid was modeled as a random walker making a fixed step of length $a = 0.1\,\mu$m. To determine the step direction, we sampled the local potential for a random walker at a given point $\mathbf{r}$ by making $n_{\mathrm{test}} = 5$ random test steps $\mathbf{r}_i$ with uniform probability around a circle of radius a centered at point $\mathbf{r}$. The potential was evaluated for each of the test steps from the free energy landscape via $U_i = F_{\mathrm{CM}}(\mathbf{r}_i)$, and then a partition function:

$$z_{test} = \sum_{i=1}^{n_{test}} e^{-\frac{U_i}{k_B T}} \tag{3}$$

is constructed. The test step for the proceeding simulation move is selected with probability $P_i = e^{-\frac{U_i}{k_B T}}/z_{test}$, which ensures that the resulting equilibrium walker position distribution $P_{\mathrm{walker}}(\mathbf{r})$ is Boltzmann distributed (i.e. $P_{\mathrm{walker}}(\mathbf{r}) \sim e^{-\frac{U(\mathbf{r})}{k_B T}}$). For each cavity eccentricity, we perform 250 runs with 3000 steps. The simulated MSD is shown below:

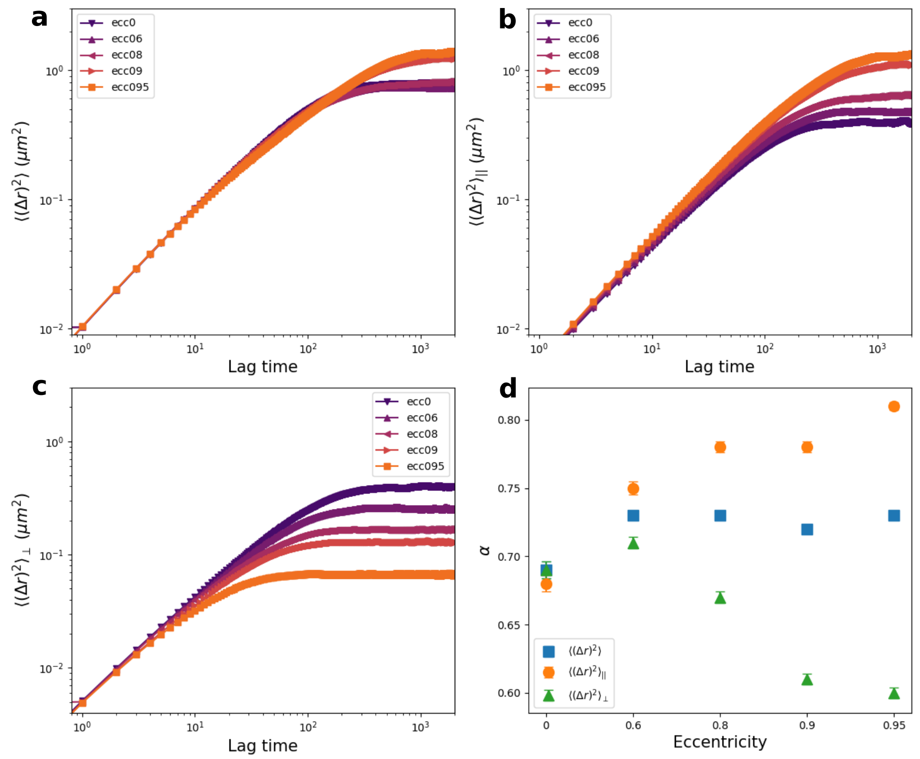

Supplementary Fig. 8: Ensemble-averaged MSD of a random walker moving in a 2D free energy landscape $F_{\mathrm{CM}}(\mathbf{r})$. **a**. The full MSD of the 2D random walker. **b**. The MSD along the cavity major-axis. **c**. The MSD along the cavity minor-axis. **d**. Scaling exponents extracted from the Brownian dynamics simulation. The blue squares indicate the exponent for the full MSD. The orange solid circles indicate the scaling exponent for the major-axis MSD component. The green triangles indicate the exponent for the minor-axis MSD component. The error bar shows the standard-error of the mean of the fitted α over $n = 250$ clips with 3000 steps.

Scaling exponents of the simulated MSD are extracted from the Monte-Carlo (MC) simulation. To ensure that the time range fitted in simulation matches to the time range fitted in experiment ($\sim$ 1 s), we calibrate our simulation time-scale by matching the simulated MSD times to the experimental result (Fig.7). We set the lag time at the point where the simulated MSD reaches 90% of its saturated value equal to the experimental lag time at the same percentage of the saturated value. The calibrated MSD plots and the corresponding exponent fitting is shown below:

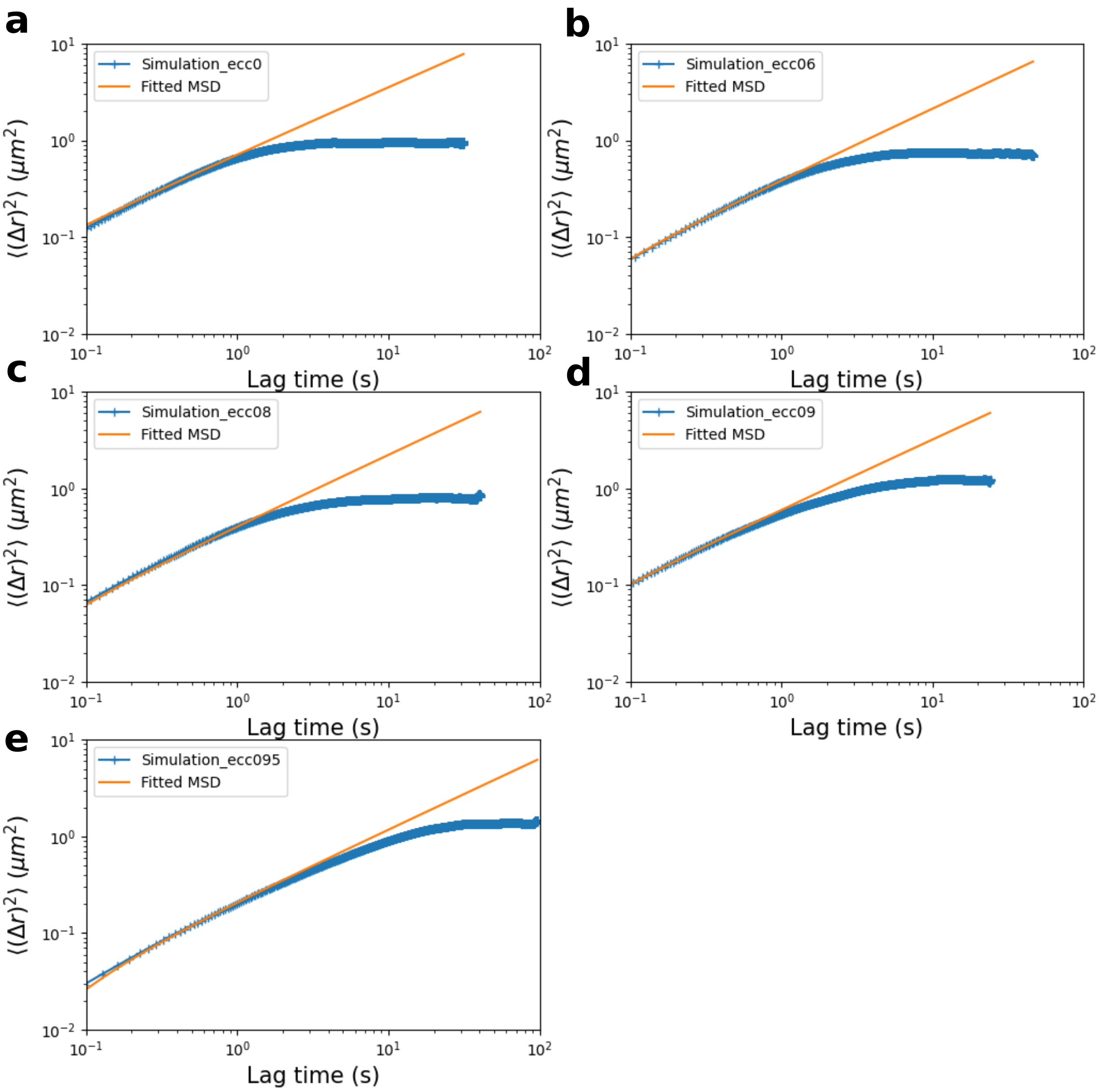

Supplementary Fig. 9: MSD of the MC simulation after time-scale calibration (blue lines) and the corresponding fit (orange lines) to extract the diffusion exponent. The fit to simulation is performed over the same short-time regime ($\leq 1\,\mathrm{s}$) as the fit against the experimental MSD. Panels **a**–**e** correspond to MSD of the MC simulation for cavities with eccentricity $e = 0$ to $e = 0.95$.

The MSD extracted from the MC simulation has a scaling exponent smaller than unity, a sub-diffusive behavior which is observed in our experiment (See Fig. 7**d**, Fig. 8**d**). We observe that the scaling exponents from the simulation share the same trend of the experimental value. Specifically, $\alpha^{\mathrm{MC}}_{\mathrm{major}}$ increases as the increasing of the eccentricity while $\alpha^{\mathrm{MC}}_{\mathrm{minor}}$ decreases as the increasing of the eccentricity. This suggests that the sub-diffusivity is largely a geometrical effect arising from the non-uniform potential landscape created by the T_4-DNA. However, the exponents from the simulation are larger than the experimental value, particularly for the perpendicular MSD component, indicating that the experiment may be uncovering dynamic behaviour that does not result purely from the particular structure of the free energy landscape. Potentially, this discrepancy arises from how the plasmid diffusion is altered by the local polymer network created by the T_4-DNA molecule, which could alter the plasmid friction factor when the plasmid transits regions of high polymer concentration, such as experienced when the plasmid crosses the cavity central axis.

In addition, to understand the origin of the two time-scales in Fig. 6 of the manuscript, we perform an equivalent dwell-time analysis on the data generated by the Brownian dynamics simulation. The result is shown below:

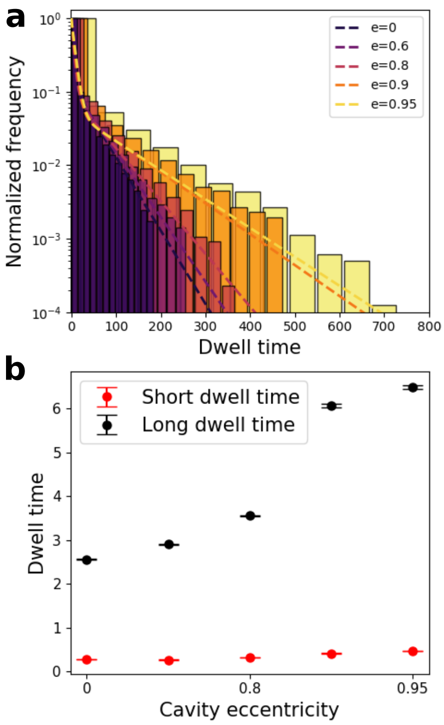

Supplementary Fig. 10: The dwell time analysis for the brownian dynamics simulation data. **a**. Dwell time histograms of the 2D random walker for cavities of varying eccentricity with double-exponential fits. **b**. Resulting average dwell-times extracted from double exponential fits to the dwell-time histograms, with the black circles corresponding to the long average dwell-time and the red circles corresponding to the shorter average dwell-time. The errorbars are extracted from the fitting covariance.

The qualitative similarity of our experimental results and simulation suggests that the distinct short and long dwell times result from the particular form of the potential landscape.

Supplementary Note 8

The device is fabricated by a three-step lithograph process illustrated in Fig. 11. The elliptical cavity is defined by an ebeam lithography procees followed by an RIE process. ZEP520A resist is spun and a thin chromium discharge layer is sputtered. Then the wafer is exposed. After exposure, the chromium layer is removed by chromium etchant and the resist is developed. We use an RIE recipe based on etch parameters suggested by Goyal et al.[1] with which we can produce smooth borofloat surfaces with R_a less than 5% of the etching depth. Microchannel is transferred to the borofloat substrate by the UV lithography process. S1818 photoresist is applied to the substrate, where the elliptical cavities are printed. A contact UV exposure followed by RIE defines the microchannel. The etched borosilicate wafer is bonded to a silicon wafer containing a 100 nm LPCVD silicon nitride film (ordered from the Cornell Nanofabrication facility). The last UV lithograph process with RIE is used to define the access window on the silicon nitride. Lastly, silicon is etched alway in KOH solution to release the free-standing membrane in the device center.

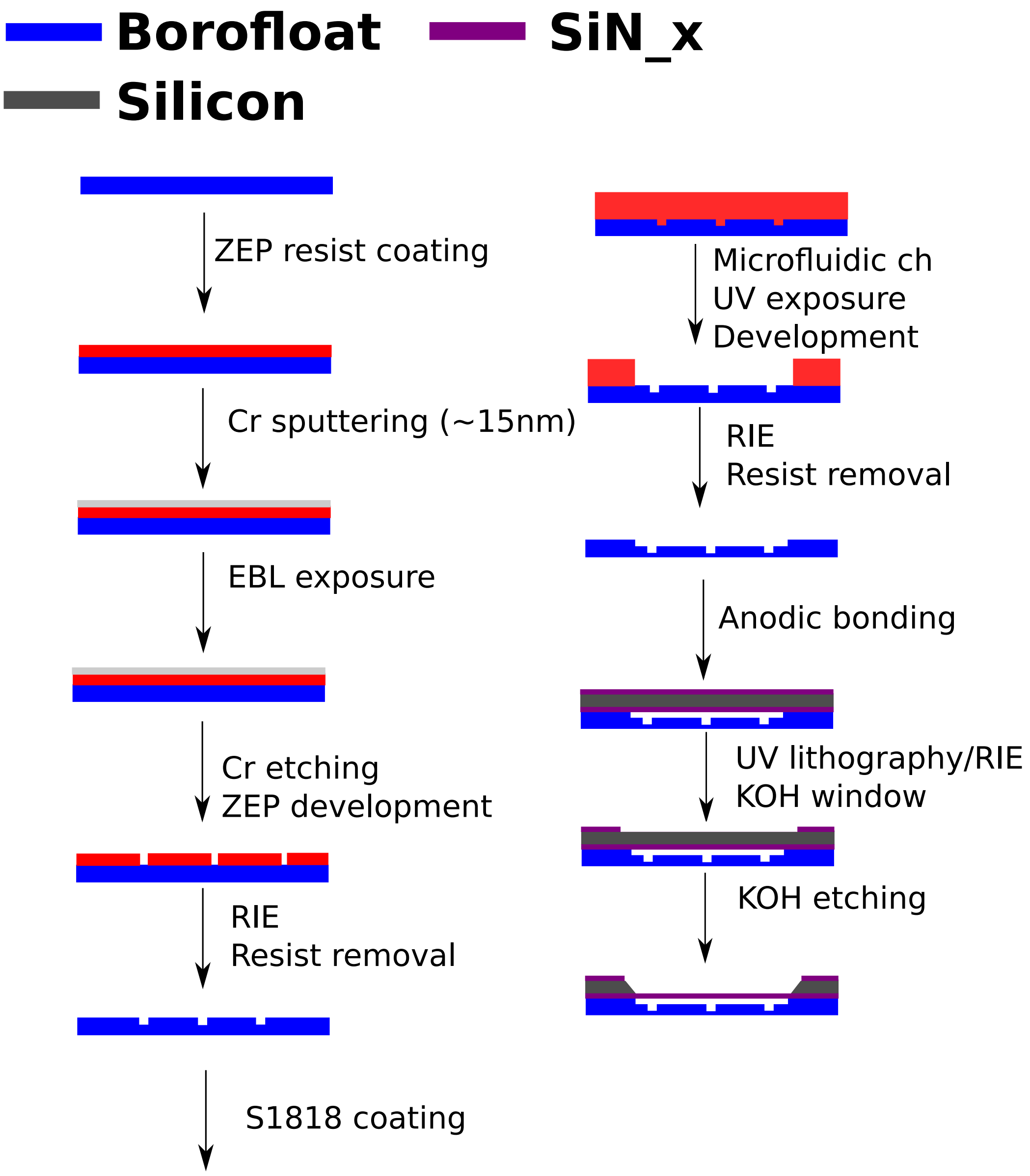

Supplementary Fig. 11: Device fabrication work flow. The device dimensions are exaggerated for clarity.

Supplementary References

[1] Abhijat Goyal, Vincent Hood, and Srinivas Tadigadapa. "High speed anisotropic etching of Pyrex for microsystems applications". In: *Journal of non-crystalline solids* 352.6-7 (2006), pp. 657–663.

[2] Yue Qi et al. "Compression of Nanoslit Confined Polymer Solutions". In: *Macromolecules* 51.2 (2018), pp. 617–625.

[3] Pauli Virtanen et al. "SciPy 1.0: fundamental algorithms for scientific computing in Python". In: *Nature methods* 17.3 (2020), pp. 261–272.

[4] John D Weeks, David Chandler, and Hans C Andersen. "Role of repulsive forces in determining the equilibrium structure of simple liquids". In: *The Journal of chemical physics* 54.12 (1971), pp. 5237–5247.
