# Supplementary material for: Confinement anisotropy drives polar organization of two DNA molecules interacting in a nanoscale cavity
Source: Nat Commun. 2022 Jul 28;13:4358. doi: 10.1038/s41467-022-31398-x (PMC9334635; doi:10.1038/s41467-022-31398-x)
Supplement: Supplementary file 3 — Reporting Summary [file 41467_2022_31398_MOESM3_ESM.pdf]

nature portfolio

Corresponding author(s): Walter Reisner
Last updated by author(s): May 30, 2022

Reporting Summary

Nature Portfolio wishes to improve the reproducibility of the work that we publish. This form provides structure for consistency and transparency in reporting. For further information on Nature Portfolio policies, see our Editorial Policies and the Editorial Policy Checklist.

Statistics

For all statistical analyses, confirm that the following items are present in the figure legend, table legend, main text, or Methods section.

n/a	Confirmed	
☐	☒	The exact sample size (*n*) for each experimental group/condition, given as a discrete number and unit of measurement
☒	☐	A statement on whether measurements were taken from distinct samples or whether the same sample was measured repeatedly
☒	☐	The statistical test(s) used AND whether they are one- or two-sided<br>*Only common tests should be described solely by name; describe more complex techniques in the Methods section.*
☒	☐	A description of all covariates tested
☒	☐	A description of any assumptions or corrections, such as tests of normality and adjustment for multiple comparisons
☐	☒	A full description of the statistical parameters including central tendency (e.g. means) or other basic estimates (e.g. regression coefficient) AND variation (e.g. standard deviation) or associated estimates of uncertainty (e.g. confidence intervals)
☒	☐	For null hypothesis testing, the test statistic (e.g. *F*, *t*, *r*) with confidence intervals, effect sizes, degrees of freedom and *P* value noted<br>*Give P values as exact values whenever suitable.*
☒	☐	For Bayesian analysis, information on the choice of priors and Markov chain Monte Carlo settings
☒	☐	For hierarchical and complex designs, identification of the appropriate level for tests and full reporting of outcomes
☒	☐	Estimates of effect sizes (e.g. Cohen's *d*, Pearson's *r*), indicating how they were calculated

Our web collection on statistics for biologists contains articles on many of the points above.

Software and code

Policy information about availability of computer code

Data collection	Micro-Manager 2.0, FIJI ImageJ2, FreeFEM
Data analysis	Customized script based on Python 3.8. The codes are available on https://github.com/echolzz/nanocavity_coderepo.

For manuscripts utilizing custom algorithms or software that are central to the research but not yet described in published literature, software must be made available to editors and reviewers. We strongly encourage code deposition in a community repository (e.g. GitHub). See the Nature Portfolio guidelines for submitting code & software for further information.

Data

Policy information about availability of data

All manuscripts must include a data availability statement. This statement should provide the following information, where applicable:

- Accession codes, unique identifiers, or web links for publicly available datasets
- A description of any restrictions on data availability
- For clinical datasets or third party data, please ensure that the statement adheres to our policy

The data that support the findings of this study are available from the corresponding author upon reasonable request.

Field-specific reporting

Life sciences study design

All studies must disclose on these points even when the disclosure is negative.

Sample size	We have a minimum of 2 videos (on average 8 videos) for each experiment scenario with each video correponding to a trapping event. The length of each video is around 5 minutes, which is long compared with the time for the confined DNA chains exploring the cavity (on the order of 10 seconds depending on the eccentricity). The total time of the video allows at least 100 pole swapping events. Figure 3c and Figure 6a show that this degree of statistics is sufficient to give rise to a well defined dwell time distribution.
Data exclusions	Videos with strong photobleaching and cross-talk are excluded.
Replication	We captured multiple videos for each experimental scenario to verify the phenomena presented in the manuscript. All the attempts are successful and the observed phenomena can be reproduced.
Randomization	The nanofluidic cavities are defined with ~10 nm precision. All the DNA samples used in this study are pure and commercially available. The experimental setup is well-defined without unknown parameters.
Blinding	Our experiment is not a randomized controlled trail with participants. Therefore blinding is not applicable.

Reporting for specific materials, systems and methods

We require information from authors about some types of materials, experimental systems and methods used in many studies. Here, indicate whether each material, system or method listed is relevant to your study. If you are not sure if a list item applies to your research, read the appropriate section before selecting a response.

Materials & experimental systems

n/a	Involved in the study	
☒	☐	Antibodies
☒	☐	Eukaryotic cell lines
☒	☐	Palaeontology and archaeology
☒	☐	Animals and other organisms
☒	☐	Human research participants
☒	☐	Clinical data
☒	☐	Dual use research of concern

Methods

n/a	Involved in the study	
☒	☐	ChIP-seq
☒	☐	Flow cytometry
☒	☐	MRI-based neuroimaging
